# Crizotinib治疗ALK阳性伴骨髓转移的肺腺癌1例及文献复习

**DOI:** 10.3779/j.issn.1009-3419.2015.02.06

**Published:** 2015-02-20

**Authors:** 晓燕 李, 晓晴 刘, 芳 高, 晓丹 尹

**Affiliations:** 100071 北京，中国人民解放军第三O七医院肺部肿瘤科 Department of Lung Cancer, 307 Hospital of PLA, Affiliated to Academy of Military Medical Sciences, Beijing 100071, China

**Keywords:** Crizotinib, 肺肿瘤, 骨髓转移, 客观有效率, 无进展生存期, Crizotinib, Lung neoplasms, Bone marrow metastasis, Objective response rate, Progression free survival

## Abstract

**背景与目的:**

肺癌患者常伴远处转移，其中伴骨髓转移患者治疗手段有限，预后差。Crizotinib已被证实可用于治疗间变淋巴瘤激酶（anaplastic lymphoma kinase, ALK）阳性的肺腺癌，但对于骨髓转移癌的疗效如何罕见报道。本文总结1例crizotinib治疗ALK阳性肺腺癌伴骨髓转移患者，并对其有效性及安全性进行讨论和分析。

**方法:**

采用原位免疫荧光杂交法（fluorescence *in situ* hybridization, FISH）检测*ALK*融合基因阳性，一线化疗失败后给予crizotinib治疗，250 mg，2次/d。按照实体瘤疗效评价标准1.1版（Response Evaluation Criteriation in Solid Tumours, RECIST v1.1）评价客观疗效，采用骨髓活检评价骨髓转移瘤疗效。按照不良反应通用术语标准4.0版（Common Terminology Criteria for Adverse Events v4.0, CTC AE v4.0）评估用药期间发生的不良事件。

**结果:**

该患者服用crizotinib 6周后，总体疗效评价为部分缓解（partial response, PR），骨髓疗效为完全缓解（complete response, CR）。因出现肺炎停药，无进展生存期（progression-free survival, PFS）20周，总生存期（overall survival, OS）22周。

**结论:**

Crizotinib治疗ALK阳性的肺癌伴骨髓转移患者可达骨髓完全缓解，耐受性好。

## Abstract (en)

**Background and objective:**

Distant metastasis was common in lung cancer, especially patients with bone marrow metastasis had poor prognosis and there were few effective methods. Crizotinib had been confirmed to be used in anaplastic lymphoma kinase (ALK) positive lung adenocarcinoma, but the efficacy in lung cancer with bone marrow metastasis was unknown. In the present study, we reported one case of ALK-positive lung adenocarcinoma with bone marrow matastasis given crizotinib treatment, the safety and efficacy was summarized.

**Methods:**

ALK fusion was tested by fluorescence *in situ* hybridization (FISH). Crizotinib was given with dose of 250 mg, *bid*. Objective response was evaluated by Response Evaluation Criteriation in Solid Tumours (RECIST) v1.1 and bone marrow response was evaluated by bone marrow puncture and biopsy. Adeverse events were evaluated according to Common Terminology Criteria for Adverse Events (CTC AE) v4.0.

**Results:**

The patient achieved partial response (PR) after 6 weeks of crizotinib, especially the objective response of bone marrow metastasis was complete response (CR). The patient stopped crizotinib because of pneumonia. The progression free survival (PFS) and overall survival (OS) was 20 weeks and 22 weeks respectively.

**Conclusion:**

Crizotinib could be an effective method for ALK-positive lung cancer with bone marrow metastasis and showed good tolerance.

肺癌是中国乃至全球发病率和死亡率最高的肿瘤之一^[1]^，易发生远处转移，常见转移部位为脑、肝、骨、肾上腺、肺等，骨髓转移较为少见，且出现骨髓转移后，常为病程后期，治疗手段有限，预后极差。分子靶向药物的临床应用为伴骨髓转移的肺癌患者带来了新的治疗思路。本文报道了1例间变淋巴瘤激酶（anaplastic lymphoma kinase, ALK）阳性伴骨髓转移的晚期肺腺癌患者，经ALK抑制剂crizotinib（克唑替尼，Xalkori，赛可瑞）治疗后，客观疗效达部分缓解（partial response, PR），且骨髓完全缓解。通过文献复习，对其安全性和有效性进行分析和讨论。

## 1 临床资料

患者，女性，40岁，因“头痛、乏力、左锁骨上肿物进行性增大1个月”于2013年10月在外院就诊，行支气管镜检查并活检，病理回报：（左固有上叶粘膜）低分化腺癌。血常规示：白细胞5.8×10^9^/L，血红蛋白102 g/L，血小板48×10^9^/L，给予重组人促血小板生成素（recombinant human thrombopoietin, TPO）治疗后血小板无明显升高，且呈进行性下降，遂行骨髓穿刺及活检，结果示“骨髓取材、涂片、染色良好，增生活跃，M=73.5%，E=17.5%，M:E=4.2:1。粒系各阶段比例及形态大致正常，红系各阶段比例及形态大致正常，红细胞轻度大小不等，中心淡染区扩大。淋巴细胞比例稍低，形态正常。单核细胞比例形态正常，全片共计数巨核细胞3个，血小板少见，活检提示骨髓组织中见低分化癌侵润，免疫组化：CK7（+）、TTF-1（+）、CK20（-），考虑来源于肺”（图 1A）。完善相关分期检查后明确诊断为：左肺腺癌（T4N3M1，Ⅳ期），多发淋巴结转移（肺门、纵隔、双侧锁骨上、右侧颈部），脑转移，骨转移，肝转移，胰腺转移，骨髓转移。突变扩增阻滞系统（amplification refractory mutation system, ARMS）（厦门艾德ADx-ARMS）检测表皮生长因子受体（epidermal growth factor receptor, *EGFR*）基因18、19、20、21外显子未见突变。分离探针FISH法（Abbott，Vysis ALK Apart FISH）检测EML4-ALK融合（+）（图 2）。

**1:**
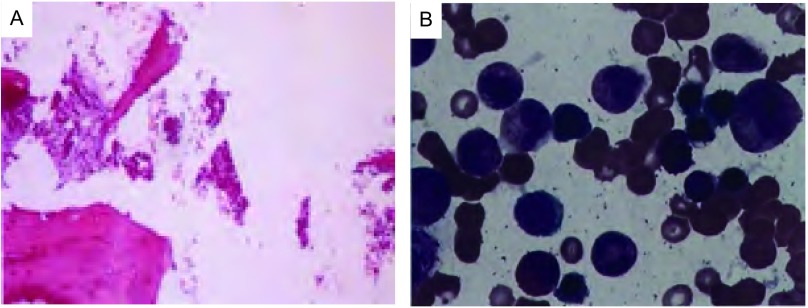
患者crizotinib治疗前后的骨髓象。A：crizotinib治疗前，骨髓组织中见低分化癌侵润，结合病史及免疫组化考虑来源于肺；B：crizotinib治疗6周后，骨髓组织中未见异常细胞。 Patient's myelogram before and after treatment of crizotinib. A: Before given crizotinib theraphy, low-dif ferentiated adenocarcinoma cell infliated in b one marrow tissue and considere d coming f rom lung according to the IHC results; B: Af ter 6 wk of crizotinib, the adenocarcinoma cell disappeared. IHC: immunohistochemistry.

**2:**
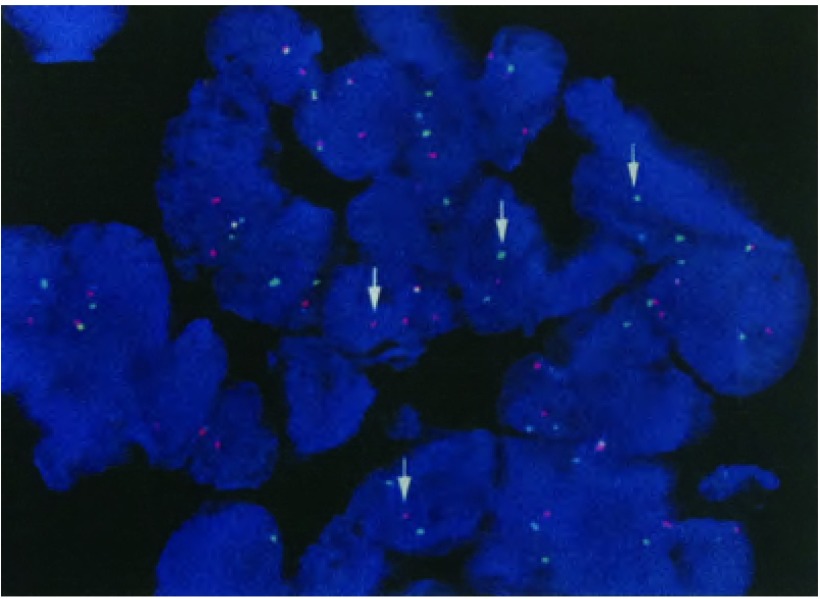
FISH法检测*ALK*融合基因阳性 *ALK* gene fusion was positive tested by FISH. ALK: anaplastic lymphoma kinase; FISH: fluorescence *in situ* hybridization.

治疗经过：患者于2013年10月10日开始全脑放疗（Dt 40 Gy/20 f/4 w），局部疗效PR。2013年11月2日给予一线化疗：培美曲噻500 mg/m^2^，d1。3周后复查胸部CT评价疗效，提示病情进展（progressive disease, PD），血常规示：白细胞3.5×10^9^/L，血红蛋白88 g/L，血小板25×10^9^/L。于2013年11月26日入我院，考虑其ALK阳性，于11月29日开始口服crizotinib，250 mg，*bid*。6周后按照实体瘤疗效评价标准1.1版（Response Evaluation Criteriation in Solid Tumours v1.1, RECIST v1.1）首次评价客观疗效，肺部疗效PR（图 3A、图 3B），颅内疗效PR，腹腔转移灶PR。血常规示：白细胞6.3×10^9^/L，血红蛋白113 g/L，血小板135×10^9^/L。再次行骨髓穿刺及活检，未见肿瘤细胞，提示骨髓完全缓解（complete response, CR）（图 1B），服用crizotinib期间，主要不良反应为乏力（1级），低蛋白血症（1级），ALT/AST升高（1级）。其后四次随访，均维持PR状态，血常规在正常范围。患者于服药20周时出现重症肺炎，停药并给予积极抗感染等内科治疗，效果欠佳，先后出现Ⅰ型及Ⅱ型呼吸衰竭，给予机械通气仍不能改善呼吸功能，于2014年5月4日死亡，自服用crizotinib起计算总生存期22周。

**3:**
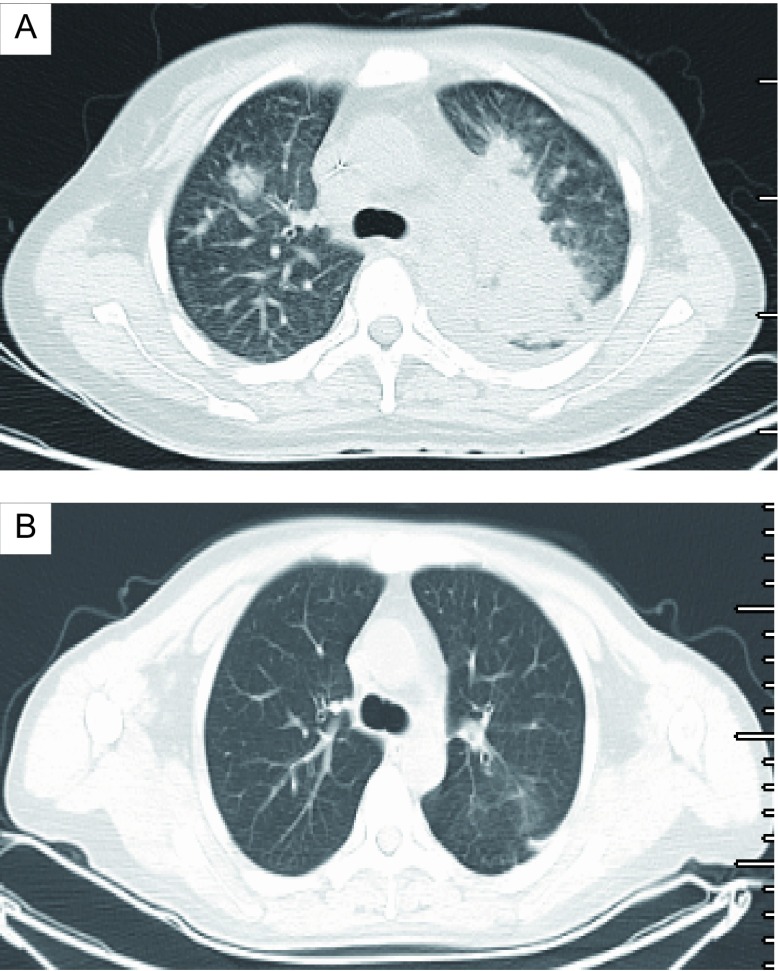
患者临床影像学特征。A：患者接受crizotinib治疗前，胸部CT显示左肺巨大占位，少量胸腔积液，右肺上叶可见团片状阴影，纵隔内可见肿大淋巴结；B：患者接受crizotinib治疗6周后，复查胸部CT显示左肺巨大占位明显缩小，胸腔积液消失，右肺上叶片状影消失，纵隔内淋巴结明显缩小。 Clinical radiologic features of the patient. A: Before crizotinib treatment, chest CT showing huge mass lesion in left lung and minimal pleural ef fusion, patchy shadows was seen in upper lobe of right lung and lymphonodus in mediastinum; B: After 6 wk of crizotinib, CT showing obvious shrink of left lung mass and lymphonomus in mediastinum, disappeared of pleural effusion and shadow in right lung. CT: computed tomography.

## 2 讨论

肺癌是全球发病率及死亡率最高的恶性肿瘤之一，其中85%为非小细胞肺癌（non-small cell lung caner, NSCLC）^[2]^，易出现远处转移，主要转移部位为骨、脑、肝、肾上腺等，但骨髓转移相对少见，总体而言，出现骨髓转移常意味着治疗效果不佳，预后极差，总生存期约3个月。

对于骨髓转移的肺癌患者，因出现严重骨髓抑制，造血功能不良，表现为外周血三系细胞减少，因此，需加强对症支持治疗，如注射粒细胞刺激因子、促血小板生成素等，或进行输血治疗。考虑骨髓抑制由肿瘤侵犯所致，上述治疗只能短暂提升三系细胞水平，因此需放疗、化疗的干预。既往报道显示，化疗药物治疗骨髓转移的疗效在小细胞肺癌（small cell lung cancer, SCLC）和NSCLC不同，SCLC骨髓转移患者对化疗较敏感，Asai^[3]^报道1例低剂量氨柔比星治疗SCLC骨髓转移达完全缓解。而NSCLC患者常因骨髓转移致外周血三系细胞减少，无化疗适应症，因此，少见化疗有效的报道。

ALK阳性肺癌是NSCLC的重要亚型，约占NSCLC的5%^[4-6]^，而在*EGFR*、*KRAS*、*HER2*或*TP53*等基因未发生突变的NSCLC患者中，ALK融合阳性的比例达25%^[6, 7]^，部分报道中*EGFR*、*KRAS*均为野生型的腺癌中ALK阳性比例高达30%-42%^[8, 9]^。Crizotinib是第一个口服的小分子ALK酪氨酸激酶抑制剂，先后进行的Ⅰ期、Ⅱ期、Ⅲ期临床研究证实了crizotinib治疗患有*ALK*融合或基因倒位的晚期NSCLC患者的安全性和有效性^[10-12]^，结果显示，ALK阳性的NSCLC患者，给予crizotinib 250 mg，*bid*，其有效率达65.3%，中位PFS 9个月，2年生存率55%。鉴于此结果，美国国立综合癌症网络（National Comprehensive Cancer Network, NCCN）指南推荐^[13]^，对于ALK阳性的NSCLC，首先建议给予crizotinib治疗，无论是一线治疗还是二线、三线或三线以后的治疗。更为重要的是，因为ALK阳性NSCLC是第一个明确靶点后再发现相应药物治疗的肺癌亚型，加之积累了EGFR-TKIs治疗肺癌的经验，此类患者治疗全程管理的重要性不言而喻^[14, 15]^。

但在临床实践中，对于伴有骨髓转移的ALK融合型NSCLC，crizotinib治疗的有效性和安全性未见报道。在本病例中，患者发病时伴多处器官转移，骨髓为受侵器官之一，相关症状表现为：恶性贫血（最低Hb 68 mg/mL）、血小板减低（最低15×10^9^/L）、重度乏力。考虑到标准铂二联化疗风险大，无法进行，故一线化疗采用培美曲塞二钠单药方案，未能控制病情。因FISH法检测ALK阳性，在一线化疗失败后开始口服crizotinib，6周后复查客观疗效，评价为PR，同时再次骨穿，证实骨髓局部疗效为完全缓解。患者共服药5个月，期间肿瘤控制稳定，未进展，无新发病灶，外周血检查在正常范围，耐受性好，体力状况明显好转，主要不良反应为crizotinib相关的轻度ALT/AST升高，无恶心、呕吐、视觉障碍等。

患者在服药19周时出现重症肺炎，经积极治疗效果欠佳，死于呼吸衰竭。复习文献可知，目前尚无crizotinib相关肺炎的报道，因此不能确定肺炎是否与crizotinib有关，仍需密切关注大样本临床用药数据。

该病例提示我们，crizotinib可随血行到达骨髓微环境，发挥局部抗肿瘤作用，较化疗药物有显著优势，所以针对ALK阳性伴骨髓转移的肺腺癌患者，crizotinib成为首选治疗方案。这是首次报道crizotinib治疗肺腺癌伴骨髓转移有效的病例。
